# Welcome message from the organizers

**DOI:** 10.1186/s42162-021-00169-1

**Published:** 2021-09-13

**Authors:** Anke Weidlich, Dirk Neumann, Philipp Staudt, Gunther Gust, Mirko Schäfer

**Affiliations:** 1 INATECH, University of Freiburg, Emmy-Noether-Str. 2, Freiburg, 79110 Germany; 2 Chair for Information Systems Research, University of Freiburg, Rempartstr. 16, Freiburg, 79098 Germany; 3 IISM, KIT, Kaiserstrasse 89, Karlsruhe, 76131 Germany

Dear readers,

The European Union intends to reduce its emissions by 55% in 2030 and aims to be climate neutral in 2050. This makes the integration of intermittent renewable power generation a question of growing importance. Simultaneously, new and potentially flexible loads are added, both large scale with electrolyzers that produce vast amounts of green hydrogen, and small scale, with millions of electric vehicles and heat pumps. Balancing demand and supply across all the diverse new loads and generators is most efficiently managed through information and communication technology, along with smart coordination methods and algorithms.

The objective of the DACH+ conference series on Energy Informatics is to promote research, development and implementation of information and communication technologies in the energy domain, and to foster the exchange of ideas and experiences between academia, industry and service providers in the German-Austrian-Swiss region and its neighbouring countries (DACH+). Over the past decade, it has established as the premier European conference in the field. Despite the challenges resulting from the COVID-19 pandemic, this year it has attracted a remarkable number of high-quality contributions. The Technical Programming Committee gave feedback on the submissions through more than 100 reviews and comments, resulting in an acceptance of 14 full and short papers, as well as a series of extended abstracts for posters and demos.

This year’s conference is organized by the University of Freiburg with support from the Fraunhofer Institute for Solar Energy Systems (ISE). Funding was provided by the German Federal Ministry for Economic Affairs and Energy. We are happy to present the proceedings of the 10th DACH+ Conference on Energy Informatics, which is held online in September 2021.

Sincerely,

Anke Weidlich (General Chair)

Dirk Neumann, Philipp Staudt, Gunther Gust (Technical Program Chairs)

Mirko Schäfer (Publication Chair)

## Data Availability

Not applicable

